# *Rickettsia* Deregulates Genes Coding for the Neurotoxic Cell Response Pathways in Cerebrocortical Neurons In Vitro

**DOI:** 10.3390/cells12091235

**Published:** 2023-04-25

**Authors:** Martin Cente, Monika Danchenko, Ludovit Skultety, Peter Filipcik, Zuzana Sekeyova

**Affiliations:** 1 Institute of Neuroimmunology, Slovak Academy of Sciences, Dubravska cesta 9, 845 10 Bratislava, Slovakia; 2 Department of Rickettsiology, Biomedical Research Center, Slovak Academy of Sciences, Dubravska cesta 9, 845 05 Bratislava, Slovakia

**Keywords:** infection, neuron, pathway, rickettsiae, signaling

## Abstract

Rickettsial infections of the central nervous system (CNS) are manifested by severe neurological symptoms and represent a serious life-threatening condition. Despite the considerable health danger, only a few studies have been conducted focusing on the pathogenesis induced by *Rickettsia* sp. in CNS. To investigate the signaling pathways associated with the neurotoxic effects of rickettsiae, we employed an experimental model of cerebrocortical neurons combined with molecular profiling and comprehensive bioinformatic analysis. The cytopathic effect induced by *Rickettsia akari* and *Rickettsia slovaca* was demonstrated by decreased neuronal viability, structural changes in cell morphology, and extensive fragmentation of neurites in vitro. Targeted profiling revealed the deregulation of genes involved in the neuroinflammatory and neurotoxic cell response pathways. Although quantitative analysis showed differences in gene expression response, functional annotation revealed that the biological processes are largely shared between both *Rickettsia* species. The identified enriched pathways are associated with cytokine signaling, chemotaxis of immune cells, responses to infectious agents, interactions between neurons, endothelial and glial cells, and regulation of neuronal apoptotic processes. The findings of our study provide new insight into the etiopathogenesis of CNS infection and further expand the understanding of molecular signaling associated with neuroinvasive *Rickettsia* species.

## 1. Introduction

Bacterial infection represents a major cause of suspected encephalitis or meningitis, with high mortality rates [1]. However, current diagnostics can identify about half of all central nervous system (CNS) pathogens [2,3].

Rickettsioses are devastating human infections [4] caused by obligate intracellular bacteria of the genus *Rickettsia*. Rickettsiae are transmitted by various arthropods (ticks, mites, lice, and fleas) and are distributed worldwide [5]. Rickettsial infections present with clinical manifestations ranging from mild febrile illness to life-threatening complications [6], including endothelial barrier dysfunction in the brain and lungs [7,8].

Acute neurological manifestations of rickettsial diseases include severe headache, behavioral abnormalities, meningismus, photophobia, amnesia, aphasia, visual and hearing loss, ataxia, seizures, encephalitis, and coma (reviewed in [9]). These symptoms are most often reported in children and patients with delayed diagnosis [10,11]. Infection and associated neuroinflammation may be fatal [12] or lead to lasting sequelae [13,14].

Neurological symptomatology in rickettsial diseases occurs after bacterial dissemination through the bloodstream and is linked with infective vasculitis [15,16]. Previous studies have confirmed rickettsial invasion of vascular endothelial cells in the brain of human patients [17,18] and experimentally infected animal models [19,20]. Rickettsiae can breach the endothelial cells of the blood–brain barrier or the epithelial cells of blood–choroid barriers and are presumably recognized by antigen-presenting cells through binding to Toll-like receptors [21,22]. Rickettsiae affect preferentially small blood vessels and cause aggregates of microglial and mononuclear cells around brain capillaries. Thrombosis of small arteries, followed by focal necrosis of the CNS, has been reported as a consequence of rickettsial infection [21,23].

Despite the considerable health danger, the molecular pathogenesis of rickettsial CNS infection is largely unknown. Moreover, in the context of etiopathogenesis of neurodegeneration, no detailed mechanisms have been described so far, although the pro-apoptotic effect of rickettsiae in neurons was demonstrated in vitro [24,25].

Using a model of primary cerebrocortical neurons, molecular profiling, and integrated bioinformatic analysis, this study aimed to characterize the underlying signaling associated with rickettsial neuroinfection.

## 2. Materials and Methods

### 2.1. Rickettsial Culture and Purification

The bacterial species used in the study were *Rickettsia akari*, strain MK (Kaplan, ATCC VR-148) isolated from a patient’s blood in New York, NY, USA [26], and *Rickettsia slovaca*, strain 13-B (ATCC VR-1639), isolated from a *Dermacentor marginatus* tick in Slovakia [27]. Rickettsiae were propagated in confluent monolayers of the Vero cell line (*Cercopithecus aethiops* kidney cells, ATCC CCL-81), maintained in an RPMI 1640 medium (Lonza, Basel, Switzerland) supplemented with 2 mM L-glutamine (Lonza, Basel, Switzerland), 25 mM HEPES (Sigma-Aldrich, St. Louis, MO, USA), and 2.5% heat-inactivated fetal bovine serum (HyClone, Logan, UT, USA) at 34 °C in a humidified 5% CO_2_ incubator. Rickettsiae were purified by a modified isopycnic density gradient centrifugation protocol described previously [28] and stored in an SPG buffer (0.218 M sucrose, 3.76 mM KH_2_PO_4_, 7.1 mM K_2_HPO_4_, and 4.9 mM potassium glutamate) at −80 °C for further experiments.

### 2.2. Infection of Primary Neurons and Viability of Host Cells

Rat cerebrocortical neurons were prepared according to a published method [29]. Briefly, the brains of anesthetized rats (18-day-old embryos) were dissected in a chilled L-15 medium free of L-glutamine (PAA, Leonding, Austria). After removal of meninges, cortical tissue was isolated, minced into small pieces, and treated with a mixture of Accutase (2 mL/10 brains) and DNAse I (1.5 ng/mL) for 10 min at 37 °C. The enzymatic treatment was stopped with fetal calf serum, and the suspension was centrifuged at 17× *g* for 3 min. The supernatant was discarded and replaced with 5 mL of culture medium. The cells were plated in 100 µL onto poly-D-lysine-coated 96-well plates at a density of 70,000/cm^2^. The medium for embryonic neurons (Neurobasal medium, Life Technologies, Carlsbad, CA, USA) was supplemented with B27 (2%), 0.5 mM L-glutamine, and Pen/Strep. Antibiotics were washed out 24 h before the experiment. Neuronal cultures were treated with a cell division inhibitor (2.5 µM cytosine β-D-arabino-furanoside) for 3–5 days in vitro to stop the proliferation of glial and non-neuronal cells. The cells were maintained in a humidified atmosphere of 5% CO_2_ at 37 °C, and the medium was replaced every 3–4 days.

Infection of primary neurons with two *Rickettsia* spp. was performed as previously reported [25]. Briefly, the number of rickettsial genome equivalents was calculated by quantitative PCR (qPCR) using genus-specific primers and TaqMan probe amplifying a portion of the *rpsL* gene as described [30]. The infectivity and quantity of purified rickettsiae were confirmed by plaque assay [31,32].

Neuron survival following infection with *Rickettsia* spp. was determined, as published previously [25]. Briefly, monolayers of cerebrocortical neurons, cultured in 96-well plates (8 × 10^4^/cm^2^) for eight days in vitro, were inoculated with *R. akari* and *R. slovaca* at a multiplicity of infection (MOI) of 1, 5, 10, and 20. After incubation at 34 °C for 24 h, the intracellular ATP was measured using CellTiter-Glo Luminescent Cell Viability Assay (Promega, Madison, WI, USA) according to the manufacturer’s instructions. The generated luminescent signal (a.u.), proportional to the amount of ATP present in cell culture, was measured using a Fluoroscan Ascent FL luminometer (MTX Lab Systems, Vienna, VA, USA). The experiment was performed in hexaplicate, and data were analyzed using Prism 9 (GraphPad Software, San Diego, CA, USA). For statistical analysis, a one-way ANOVA with Dunnett’s multiple-comparison test was performed. A *p*-value of <0.05 was considered significantly different.

For gene expression profiling, rat primary neurons cultured in 6-well plates (1 × 10^5^/cm^2^) for eight days in vitro were inoculated with *R. akari* at MOI 2.5 and 5 and *R. slovaca* at MOI 10 and 20. Following the infection for 24 h, cells were harvested from the culture plates for subsequent RNA extraction.

### 2.3. Immunofluorescence Assay (IFA)

Cell cultures of rat cerebrocortical neurons were infected with *R. akari* at MOI 5 and *R. slovaca* at MOI 20. After 36 h, IFA was performed according to a published protocol [33]. As negative controls, infected neurons were probed with secondary antibodies only, and uninfected cells treated with both primary and secondary antibodies were included. Images were acquired by a Zeiss LSM 510 META confocal fluorescent microscope. The contrast was adjusted by LSM Image Browser software (Zeiss, Jena, Germany). The final images with legends were created in CorelDRAW version 24.0.0.301 (Alludo, Ottawa, ON, Canada).

### 2.4. RNA Extraction

Total RNA was isolated using the RNeasy Mini Kit according to the manufacturer’s recommendations, including the DNase I digestion step (Qiagen, Hilden, Germany). RNA was eluted into 40 µL RNase-free water. The integrity of isolated RNA samples was determined with an Agilent 2100 Bioanalyzer using an RNA 6000 Nano Labchip kit (Agilent Technologies, Waldbronn, Germany). For qPCR analysis, high-quality RNA samples were used (RNA integrity number = 6.4 to 8.8). Synthesis of the first strand cDNA was carried out using the High Capacity cDNA Reverse Transcription Kit (Applied Biosystems, Foster City, CA, USA) according to the manufacturer’s protocol. Briefly, 10 µL of the 2× reverse transcription master mix was combined with an RNA sample (1 µg/10 µL), and cDNA was synthesized under the following conditions: 25 °C for 10 min, 37 °C for 120 min, 85 °C for 5 min, and 4 °C for 1 min.

### 2.5. Gene Expression Profiling Using Real-Time PCR

Gene expression profiling was performed using the predesigned TaqMan gene expression assays (Applied Biosystems, Foster City, CA, USA). A list of analyzed genes is provided in Supplementary Spreadsheet S1. The composition of the qPCR reaction (25 µL) was as follows: 12.5 µL 2× TaqMan gene expression master mix; 1.25 µL 20× target FAM-labelled TaqMan primer assay; 10.25 µL nuclease-free H_2_O and 1 µL cDNA sample (50 ng/µL). PCR reactions were performed in triplicates under the following conditions: 50 °C for 2 min, 95 °C for 10 min, followed by 40 cycles of 95 °C for 15 s and 60 °C for 1 min. Comparative cycle threshold (2^−ΔΔCt^) analysis was performed to compare gene expression between control (uninfected) and *Rickettsia*-infected rat primary neuronal cells using the eukaryotic 18S rRNA (Hs99999901_s1), Actin beta (Rn00667869_m1), and glyceraldehyde-3-phosphate dehydrogenase (Rn99999916_s1) as endogenous reference genes. Results are expressed as a fold change of the mRNA level in infected cells compared with uninfected control primary neurons. Genes with a fold change ≥1.5 were defined as differentially expressed.

### 2.6. Pathway Enrichment Analysis (PEA)

Human orthologs for deregulated genes were used to query the Enrichr tool with a Bioplanet 2019 pathway library (KEGG, BioCarta, Reactome, WikiPathways, NCI-Nature, Science Signaling, and NetPath databases) and Gene Ontology (GO) Biological process 2021 library (https://maayanlab.cloud/Enrichr accessed on 24 August 2022) [34]. Using the Enrichr platform, the input gene set was queried against libraries of annotated gene sets with the goal of identifying significantly overlapping genes. These genes were further annotated for their affiliation to signaling pathways (Bioplanet 2019 library) and biological processes (Gene Ontology 2021 library), and enrichment results were calculated. The bioinformatic analysis included genes with an average ≥1.5-fold up-regulation and ≤−1.5-fold down-regulation to identify significantly enriched pathways for *R. akari* (15 genes) and *R. slovaca* (11 genes) separately. Enriched pathways and GO biological processes are ranked by the q-value (adjusted p-value calculated using the Benjamini–Hochberg method, q < 0.05 considered significant) and odds ratio. To visualize both common and unique pathways and biological processes among rickettsia species, we used NAViGaTOR ver. 3.0.11 [35]. Top enriched terms are highlighted by statistical significance (pink, blue, and grey refer to low-to-higher q-value) and odds ratio (node size). Final graphs were exported in SVG format and post-processed in CorelDRAW version 24.0.0.301 to produce the final images with legends (Alludo, Ottawa, ON, Canada). A comprehensive list of enriched pathways and GO biological processes for each species is provided in Supplementary Spreadsheet S1.

## 3. Results

### 3.1. Neuronal Damage Induced by R. akari and R. slovaca

**Figure 1:**
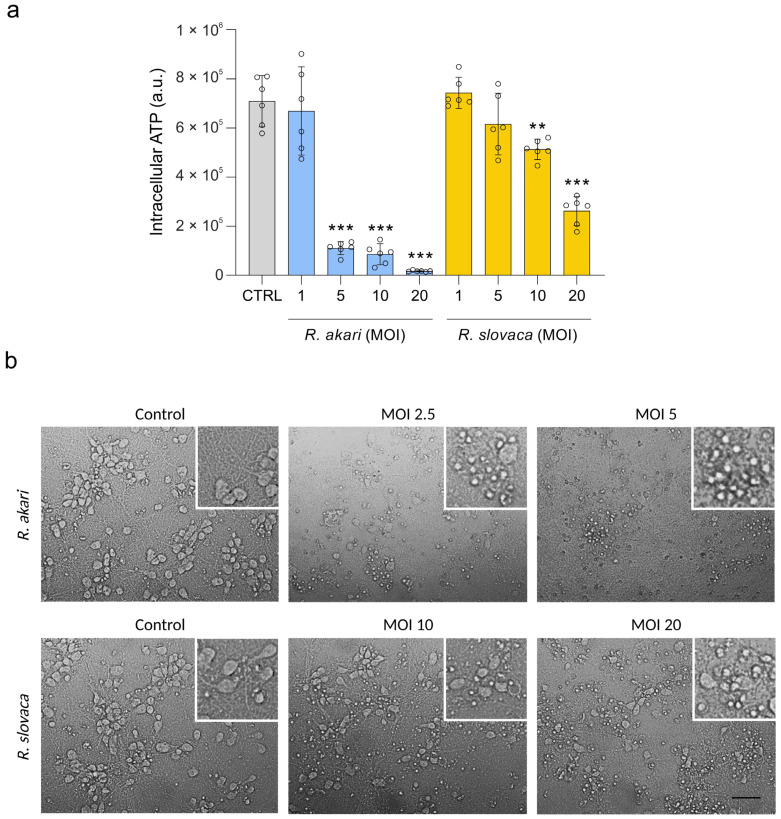
The cytopathic effect of rickettsiae in primary cerebrocortical neurons. (**a**) Primary rat neuronal cultures were incubated with *R. akari* and *R. slovaca* at MOI 1, 5, 10, and 20. Both *Rickettsia* spp. induced a statistically significant decrease in host cell viability 24 h post-infection compared to uninfected control (CTRL) neurons (one-way ANOVA, ** *p* < 0.01, *** *p* < 0.001; values are displayed as empty dots, *n* = 6, columns represent mean with 95% confidence interval). (**b**) Light microscopy of cerebrocortical primary neurons infected with *R. akari* at MOI 2.5 and 5 and *R. slovaca* at MOI 10 and 20. The cytopathic effect of rickettsiae is represented by structural changes in cell morphology, extensive fragmentation of neurites, and neuronal loss. Scale bar represents 50 μm.

The neurotoxic effect of rickettsiae was elucidated using an experimental model of rat embryonal neurons. Cerebrocortical primary neurons were infected in vitro with two different bacterial species of the genus *Rickettsia*, namely *R. akari* and *R. slovaca*. First, to observe cell culture viability in response to rickettsial infection, we measured the intracellular concentration of ATP in an inoculation dose-dependent manner. Rickettsiae induced a statistically significant decrease in the viability of neurons 24 h post-infection. In the case of *R. akari*, infection resulted in the loss of 83.7, 87.3, and 97.5% ATP in host cells when inoculated with MOI 5, 10, and 20, respectively, and profoundly compromised neuronal survival. Significant differences in cell viability were also detected after infection with *R. slovaca* at MOI 10 and 20, which resulted in the loss of 24.3 and 61.4% ATP, respectively, compared to uninfected control cultures (Figure 1a).

**Figure 2:**
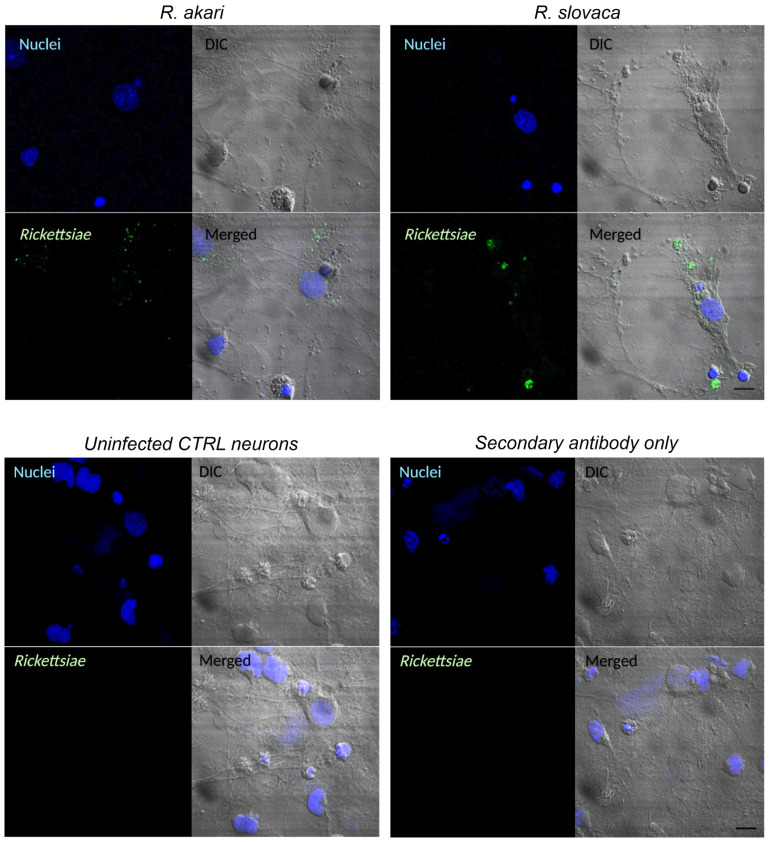
Detection of rickettsiae by indirect IFA. Infected neurons were labeled with anti-*R. akari* and anti-*R. slovaca* rabbit primary antibodies, followed by Alexa Fluor 488 conjugated goat anti-rabbit secondary antibodies (**upper** panels, green signal). No signal was detected in uninfected neurons or in control staining using only secondary antibodies (**lower** panels). Cell nuclei were stained with DAPI (blue signal). To show the overall shape of the neurons, we applied differential interference contrast (DIC). Scale bar represents 10 μm.

Based on the results of the ATP cell viability assay, we next infected the cerebrocortical neurons for gene expression profiling with *R. akari* at MOI 2.5 and 5 and *R. slovaca* at MOI 10 and 20. After 24 h post-infection, the primary neuronal culture was observed for induced cytopathic effect microscopically (Figure 1b). We recorded evident phenotypic changes in neurons after infection with both rickettsiae, showing strong destruction of cell morphology when compared to uninfected control cultures. Furthermore, we visualized *R. akari* and *R. slovaca* intracellularly in cerebrocortical neurons 36 h post-infection by IFA. Bacteria were localized inside the neurons, as well as in cell neurites (Figure 2 upper panels). Uninfected control cells labeled with both primary and secondary antibodies and infected cells probed with only secondary antibodies showed no positive signal (Figure 2 lower panels).

### 3.2. Rickettsiae Induce Deregulation of Gene Expression in Primary Cerebrocortical Neurons

**Figure 3:**
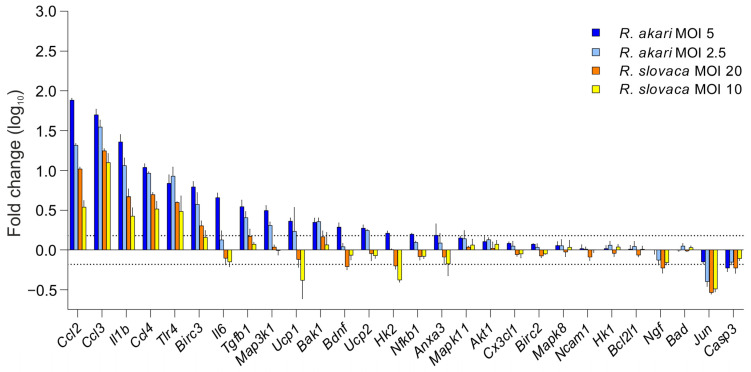
Deregulated genes in *Rickettsia*-infected neurons in vitro. Quantitative profiling revealed overexpression of pro-inflammatory and pathogen-responsive genes after *R. akari* and *R. slovaca* infection of rat-primary neurons. The neurotoxic effect of both bacterial species was also associated with the deregulation of genes involved in signal transduction, apoptosis, and cell survival. The dotted line indicates the gene expression threshold (≥1.5-fold for up-regulation and ≤−1.5-fold for down-regulation compared to uninfected controls). Columns represent the mean log_10_-fold change of gene expression in infected neurons with a 95% confidence interval (*n* = 3), and color indicates the MOI of individual *Rickettsia* species.

In order to investigate the molecular signaling associated with the neurotoxic effects of rickettsiae, we performed a targeted analysis of key regulatory genes involved in the neuroinflammatory and neurotoxic cell response pathways. Specifically, the infection of primary cortico-hippocampal neurons with *R. akari* resulted in increased expression of pro-inflammatory chemokines and cytokines (↑*Ccl2*, ↑*Ccl3*, ↑*Ccl4*, ↑*Il1b*), pathogen infection and innate immunity responsive genes (↑*Il6*, ↑*Tlr4*, ↑*Tgfb1*), deregulation of apoptosis and cell survival regulatory genes (↑*Bak1*, ↑*Bdnf*, ↑*Birc3*, ↓*Casp3*, ↑*Ucp1*, ↑*Ucp2*), and genes involved in signal transduction (↓*Jun*, ↑*Map3k1*). The toxic effect of *R. slovaca* on primary neurons was associated with the up-regulation of *Ccl2*, *Ccl3*, *Ccl4*, *Il1b*, *Tlr4,* and *Birc3* genes and down-regulation of *Ucp1*, *Hk2*, *Ngf*, *Casp3*, and *Jun*. Quantitative analysis showed differences in the gene expression response between the tested species. In particular, *R. akari* induced the up-regulation of *Bak1*, *Bdnf*, *Il6*, *Map3k1*, *Tgfb1*, and *Ucp2*, while the expression of these genes was not altered after *R. slovaca* infection. We found a specific down-regulation of *Hk2* and *Ngf* together with an opposite pattern of *Ucp1* expression in *R. slovaca* compared to *R. akari*, suggesting several distinct molecular pathways associated with neuronal toxicity of these *Rickettsia* spp. Overall, the magnitude of altered gene expression corresponded to the MOI of individual pathogen species. Moreover, *R. akari* showed a higher neurotoxic effect compared to *R. slovaca*, which was represented by a greater number of deregulated genes at lower MOI (Figure 3). Comprehensive gene expression profiles are shown in Supplementary Spreadsheet S1.

### 3.3. Signaling Pathways Involved in Response to Rickettsial Infection in Primary Cerebrocortical Neurons

**Figure 4:**
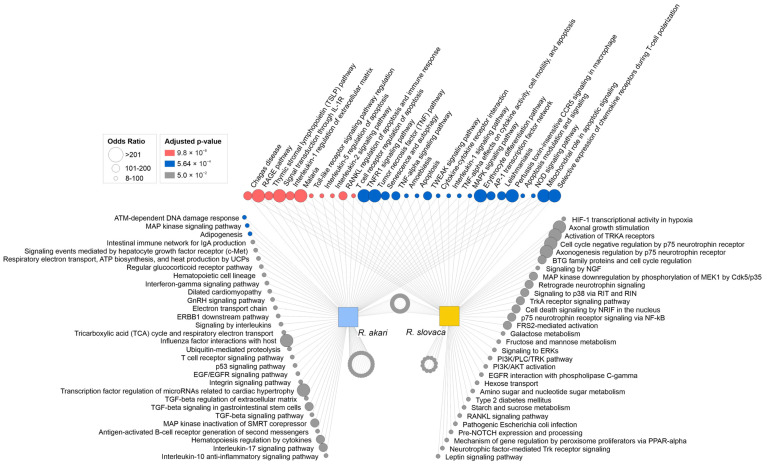
Enriched pathways associated with rickettsial infection. Pathway enrichment analysis identified a dominant neuronal response that is shared between *R. akari* and *R. slovaca*. Top over-represented enriched pathways for each species, as revealed by the Enrichr tool and the Bioplanet pathway library, are closely related to infectious disease, apoptosis, inflammation, and neuronal survival signaling. Node size corresponds to the odds ratio, and color represents the significance of pathway enrichment (pink, blue, and grey refer to low-to-higher q-value). Pathway names are listed for the top 30 enriched pathways in each group, while the grey circles represent the remaining significantly enriched pathways (152 shared pathways, 31 *R. akari*-specific pathways, 12 *R. slovaca*-specific pathways).

To elucidate the functional annotation of signaling pathways associated with rickettsia-induced neurotoxicity, we performed integrated bioinformatic analysis using the Enrichr tool with the Bioplanet 2019 pathway library. Overall, we identified 243 significantly enriched pathways for *R. akari* and 224 significantly enriched pathways for *R. slovaca*. Our analysis revealed that the majority of identified enriched pathways are shared between both rickettsiae (182 pathways), and only a fraction of enriched pathways are unique for either of the tested species (*R. akari*: 61 pathways, *R. slovaca*: 42 pathways). The group of shared enriched pathways associates with pro-inflammatory signaling via TNFα, IL-1, IL-2, and IL-5, microbial infection signaling, as well as regulation of apoptosis, senescence, and autophagy. A panel of top enriched pathways specific to *R. akari* includes DNA damage response, host–pathogen interactions, TGFβ, chemokine, and cytokine signaling. Enriched pathways specific to *R. slovaca* are mostly related to pro-apoptotic signaling, activation of programmed cell death, and suppressed cell survival pathways (Figure 4). These data are consistent with the decreased cell viability and reduced neuronal survival observed after rickettsial infection of cerebrocortical neurons in vitro.

**Figure 5:**
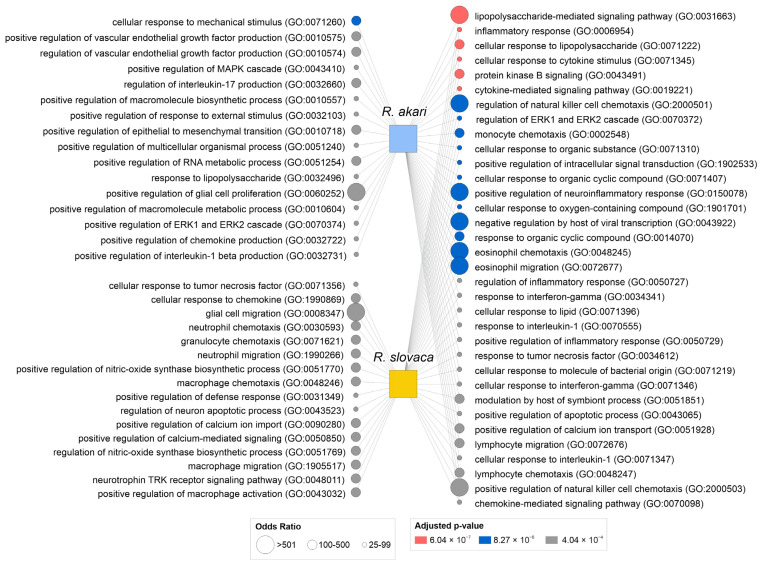
Biological response of cerebrocortical neurons to rickettsial infection in vitro. Bioinformatic analysis revealed a significant overlap of biological processes involved in neuronal signaling after infection with *Rickettsia* spp. Deregulated genes associated with neuroinflammatory response mechanisms, activation of immune and glial cells together with regulation of apoptotic processes, and host–pathogen defense responses. The scheme visualizes the top 50 significant GO biological processes for each species (34 shared processes, 16 *R. akari*-specific processes, 16 *R. slovaca*-specific processes). Node size corresponds to the odds ratio, and color represents the significance of biological process enrichment (pink, blue, and grey refer to low-to-higher q-value). The number in parentheses refers to the GO identifier.

Bioinformatic analysis linked deregulated genes to biological processes involved in the neuronal response to *R. akari* and *R. slovaca* infection in vitro. Most of the top over-represented GO biological processes are shared between both species, including dominant cellular responses to infectious agents, cytokine signaling, and chemotaxis of immune cells. Unique biological processes identified by enrichment analysis also highlighted similar neuroinflammatory responses following *R. akari* and *R. slovaca* infection, including glial cell proliferation and migration, macrophage chemotaxis, and regulation of neuronal apoptotic processes (Figure 5). A comprehensive list of GO biological processes is provided in Supplementary Spreadsheet S1.

## 4. Discussion

Neurological manifestations of rickettsioses include behavioral abnormalities, meningoencephalitis, focal neurological deficits, stroke, seizures, and coma. Interestingly, it is not clear why only a subgroup of patients, e.g., 40% of those with spotted fever rickettsioses, develops neurologic sequelae [36,37,38]. We have previously shown that rickettsial infection is associated with extensive destruction of neuronal morphology, fragmentation of neurites, and decreased viability. However, the observed difference in the cytopathic effect of tested rickettsiae was not correlated with the viability of purified bacteria, which may have influenced previously published data. The present analysis confirmed the infectivity and quantity of rickettsiae by plaque assay, providing a better characterization of isolated bacteria in comparison to previous results [25].

Here we explored two genetically different clusters of rickettsiae (*R. akari* and *R. slovaca*), aiming to characterize the specific signaling pathways associated with their neurotoxic effects. Using targeted gene profiling and comprehensive bioinformatic analysis, we identified key molecular mechanisms linked to rickettsial neuroinfection.

Our findings indicate dominant neuroinflammatory and pro-apoptotic signaling, activation of programmed cell death, and suppressed cell survival pathways in neurons after the infection. The comparison of *R. akari* and *R. slovaca* revealed that the neuronal response to these pathogens is largely shared, suggesting very similar signaling.

At the cell level, since rickettsiae are obligate intracellular bacteria, a recent hypothesis includes two ways of intrusion, direct or indirect neuroinvasion or immunologically mediated neuronal damage [9]. In spite of the clinical importance and neurological consequences of rickettsial brain infection, including neuroinflammation and neurotoxicity, very little research was performed in order to reveal underlying molecular mechanisms induced by rickettsiae in neurons. It was reported that massive activation of microglia after rickettsial infection results in neurotoxicity and inflammation of the CNS, leading to the death of infected mice [12]. However, in the context of the published data, we suppose that microglia migrate to the site of neuronal damage and exert neuroprotective processes, as this is their authentic function in a diseased brain [39]. The recent review summarizes the pathogenesis of *Rickettsia*-infected mice and presents a set of upregulated genes coding for specific cytokines and indicates affected brain cells [14].

In the present study, we confirmed that the bacteria are clearly localized inside the neuronal cell bodies and in cell neurites. As a consequence of the intraneuronal pathogenic activity of rickettsiae, we found that the identified biological processes are associated with increased migration and chemotaxis of macrophages, lymphocytes, and natural killer cells, suggesting that neuronal signaling attracts phagocytic and immune cells to sites of rickettsial neuroinfection.

The spreading of rickettsiae throughout the body leads to the infection of the vascular endothelium of the small and medium-sized blood vessels [40]. Thus, most of the clinical symptoms of rickettsial diseases are associated with the infection of endothelial cells [41]. Our analysis revealed that neuronal infection is associated with identical signaling pathways, as observed in *Rickettsia*-infected endothelial cells. In particular, NF-κB-mediated regulation of apoptotic pathways [42], represented by the balance between pro- (↑*Bak1*) and anti-apoptotic (↑*Birc3*) signals and prevention of caspase activation (↓*Casp3*) was observed. Mitochondrial role in apoptotic signaling in neurons was observed concluding the importance of this signaling as it was described in *Rickettsia*-infected endothelial cells [43]. Furthermore, infected neurons overexpressed a panel of pro-inflammatory chemokines, cytokines, and innate immunity-responsive genes, such as *Il1b*, *Il6*, *Ccl2*, *Ccl3*, and *Ccl4* that were previously reported in *Rickettsia*-infected endothelium [44]. Typical host response to bacterial infection is mediated via TLR4/lipopolysaccharide signaling that triggers pro-inflammatory reactions. Mechanisms involving TLR4 and MyD88 were previously reported in dendritic cells, playing a critical role in eradication of the invading bacteria and host protection in vivo [45,46]. Immune responses via multiple TLRs and NLRs recognizing endotoxins from *R. akari* were also documented [47,48].

Based on the data, we conclude that rickettsial neuroinfection induces innate immunity pathways involving TLRs with its downstream signals. Furthermore, the bioinformatic analysis highlighted significant crosstalk between neuronal, glial, and endothelial cells, the main components of the blood–brain-barrier. Positive regulation of vascular endothelial growth factor production, together with the regulatory pathways of migration and proliferation of glial cells, suggests that rickettsial neuroinfection may also activate reparative mechanisms necessary for the neuronal protection, re-establishment of homeostasis and integrity of the blood–brain-barrier. These pro-survival signals at the background of observed neuronal cell death in vitro are not surprising, as the rickettsiae manipulate host cell death mechanisms using several strategies (reviewed in [48])**.**

The findings of our study are consistent with published observations and further expand the understanding of molecular signaling associated with neuroinvasive *Rickettsia* species. Nevertheless, we acknowledge several limiting aspects that need to be considered. In particular, the analysis explored two specific *Rickettsia* species targeting only a subset of regulatory mechanisms that may be affected after infection of neuronal cells. Recent research in non-neuronal cells has identified impairment of many other regulatory pathways involved in *Rickettsia*–host interactions, such as cell adhesion, autophagy, cytoskeleton, oxidative stress, or regulation by non-coding RNAs [48,49], which were not investigated in more detail in this analysis. We are also aware that host–pathogen interactions may be cell-type and species-dependent, as the signaling mechanism may differ between the diverse hosts [43,50]. Therefore, further studies are warranted to explore the broad complexity of signaling pathways after rickettsial infection of neurons considering these aspects.

## 5. Conclusions

We report a focused signature of molecular signaling associated with the neurotoxic effects of *R. akari* and *R. slovaca,* highlighting a shared neuroinflammatory and pro-apoptotic signaling, activation of programmed cell death, and suppressed cell survival pathways in vitro. A complex understanding of neuronal responses may help to elucidate the pathogenesis of rickettsial neuroinfections, leading to the identification of potential prognostic markers and improving the design of treatment regimens in the future.

## Data Availability

The datasets used and analyzed during the current study are available from the corresponding authors upon reasonable request.

## Supplementary Materials

The following supporting information can be downloaded at: https://www.mdpi.com/article/10.3390/cells12091235/s1, Spreadsheet S1: Gene expression data and significantly enriched pathways after *Rickettsia* spp. infection.
